# A stable cyclized antimicrobial peptide derived from LL-37 with host immunomodulatory effects and activity against uropathogens

**DOI:** 10.1007/s00018-022-04440-w

**Published:** 2022-07-11

**Authors:** John Kerr White, Taj Muhammad, Emelie Alsheim, Soumitra Mohanty, Anna Blasi-Romero, Sunithi Gunasekera, Adam A. Strömstedt, Natalia Ferraz, Ulf Göransson, Annelie Brauner

**Affiliations:** 1 Department of Microbiology, Tumor and Cell Biology, Karolinska Institutet, 17176 Stockholm, Sweden; 2 Division of Clinical Microbiology, Karolinska University Hospital, Stockholm, Sweden; 3 Pharmacognosy, Department of Pharmaceutical Biosciences, Biomedical Centre, Uppsala University, Box 591, 75124 Uppsala, Sweden; 4 Nanotechnology and Functional Materials, Department of Materials Science and Engineering, Uppsala University, Box 35, 75103 Uppsala, Sweden

**Keywords:** Cyclized antimicrobial peptide, Urinary tract infection, *E. coli*, Urinary catheter, Innate immunity

## Abstract

The increasing antibiotic resistance among uropathogenic bacteria warrants alternative therapeutic strategies. We demonstrate the potential of the synthetic peptide CD4-PP, designed by dimerization and backbone cyclization of the shortest antimicrobial region of human cathelicidin, LL-37. CD4-PP is active against clinical and type strains of common uropathogens *Escherichia coli*, *Klebsiella pneumoniae*, and *Pseudomonas aeruginosa* at concentrations substantially below cellular cytotoxic levels and induced membrane deformation and leakage in *E. coli* and *P. aeruginosa*. Furthermore, CD4-PP treatment prevented the formation of new biofilm and dissolved mature biofilm created by *E. coli* and *P. aeruginosa* and targeted curli amyloid in *E. coli* biofilms*.* In addition, CD4-PP also induced production of LL-37 by uroepithelial cells and increased the expression of tight junction proteins claudin-14 and occludin. During uroepithelial cell infection, CD4-PP significantly reduced uropathogen survival when treatment was given at the start of infection. Low micromolar of CD4-PP treatment initiated after 2 h was successful with all tested species, except *P. aeruginosa* where CD4-PP was unable to reduce survival, which could be attributed by early biofilm formation. Finally, we demonstrated that urinary catheter pieces coated with saline fluid supplemented with CD4-PP reduced the attachment of *E. coli*, giving it a potential clinical application.

**Supplementary Information:**

The online version contains supplementary material available at 10.1007/s00018-022-04440-w.

## Table of contents

Annually, over 150 million cases of urinary tract infections (UTIs) occur, with an increasing number of multidrug-resistant strains hampering antibiotic treatment. Therefore, alternative treatment strategies are needed. The synthetic antimicrobial peptide, CD4-PP, has a direct bactericidal and immunomodulatory effect and could be a novel therapeutic strategy to prevent and treat UTIs, thereby preventing antibiotic consumption.

## Introduction

On a global perspective, urinary tract infections (UTIs) are one of the most common bacterial infections. More than 50% of women have at least one episode during their lifetime and 40% of them experience recurrent infections. Patients with urinary catheters are colonized with bacteria within weeks and run increased risk of recurrent UTIs and even life-threatening urosepticemia. The majority of all UTIs are caused by *Escherichia coli* [1] and can be treated with antibiotics, although an increasing number of UTI causative bacteria are now displaying antibacterial resistance [2]. This increase in resistance complicates the treatment with antibiotics and warrants other therapeutic strategies. We here perform a thorough chemical and biological characterization of a synthetic peptide designed from the human antimicrobial peptide (AMP) LL-37, with special focus on its potential clinical application in preventing and handling of difficult-to-treat UTIs.

The release of endogenous antimicrobial peptides (AMPs) constitutes an important component of the body’s arsenal for preventing and combating UTIs. AMPs are secreted by both immune and epithelial cells and are part of the body’s first line of defense against invading pathogens. These compounds are generally cationic and amphipathic, and possess potent bactericidal effects against common human pathogens [3, 4]. In addition they are also powerful immunomodulators, and are able to induce the production of cytokines and chemokines [5, 6], as well as tight junction proteins [7] in host cells.

In the context of UTI, AMPs such as defensins [8], the ribonuclease A superfamily [9] and LL-37, the only member of the cathelicidin family found in humans [10], are part of the innate immunity protecting the host from invading pathogens. Currently, synthetic LL-37 is being used in clinical trials treating venous leg ulcers [11, 12]. Despite the interesting bioactivities of LL-37, its direct utility in a clinical setting has been hampered due to its toxicity to human cells and proteolytic instability [13, 14]. To overcome these issues, engineering of peptides derived from LL-37 into biologically stable form(s) presents as a promising option for potential clinical application.

We have previously demonstrated that it is possible to increase the activity of the shortest antimicrobial region of LL-37, known as KR-12 [15], by substituting specific amino acids [16]. Further engineering of KR-12 derivatives into head-to-tail cyclic dimers resulted in peptides with both increased proteolytic stability and antimicrobial activity [17]. In the current study, we show that a novel cyclized dimer, known as CD4-PP, now has enhanced structural stability and elevated activity at physiologically relevant conditions. Different from previous studies where linear peptides were designed to improve peptide activity, stability and toxicity [18], this study documents anti-biofilm and in vitro efficacy of a cyclic form of LL-37 to gain stability and efficacy. In particular, we assessed whether CD4-PP is active against common uropathogens, exhibits immunomodulatory effects in uroepithelial cells, and could prevent bacterial attachment to urinary catheters as a possible clinical application.

## Methods

### Synthesis of CD4-PP

CD4-PP was synthesized as described previously [17]. In brief, a Dawson Dbz AM resin (substitution value 0.49 mmol/g, Merck) was used as a solid support and the first residue was coupled manually at room temperature. A microwave assisted Fmoc/tBu protocol on Liberty 1 peptide synthesizer (CEM Corporation) was used to elongate the sequence. The resin-bound peptide containing the Dbz linker was acylated using 4-nitrophenylchloroformate (Sigma-Aldrich) in dichloromethane (DCM), and later activated with 0.5 M DIPEA (Sigma-Aldrich) in dimethyl formamide (DMF; Saveen-Werner)). The washed and dried peptide-Nbz resin was cleaved off from the solid support using trifluoroacetic acid (TFA; Sigma-Aldrich))/trimethylsilylisopropane (TIPS)/water (95.5:2.5:2.5, v/v/v) for 3 h, under stirring, at RT. Backbone cyclization was achieved using native chemical ligation (NCL) by re-suspending Nbz-peptide in cyclization buffer for 24 h at RT [19].

The mixture containing the cyclized product was desalted by size exclusion (Sephadex G-15; GE Healthcare Lifesciences). Biotage®Sfär C18 D cartridge (Duo 100 Å 30 µm 12 g. 2/cs) columns coupled to ÄKTA Fast Protein Liquid Chromatography (FPLC) system (GE Healthcare, Sweden) with detection at 254 nm were used for large-scale purification. Subsequently, HPLC was used for final purification [Waters, Milford, MA; Xbridge Peptide BEH 300 Å, 5 µm, 250 × 10 (i.d.) mm] using a LC-20 Shimadzu system operated with a linear gradient of increasing solvent B (acetonitrile, AcN, containing 0.05% TFA) in solvent A (H_2_O containing 0.05% TFA) over 80 min at a flow rate of 3 mL/min. Peptide purity was determined using HPLC (Phenomenex Jupiter 5 µm C18 300 Å, LC column, 250 × 4.6 mm) with a linear gradient of 5–50% AcN in water, containing 0.05% TFA, at 1 mL/min over 18 min. Peptide identity was confirmed on Waters Xevo G2-XS mass spectrometer coupled with Acquity UPLC peptide BEH C18 Column (130 Å, 1.7 µm, 150 × 0.075 mm) using solvent C (0.1% FA in water) and D (0.1% FA in AcN). A linear gradient from 0 to 90% D in solvent C was used for analysis over 75 min. Reference peptides, KR-12 and LL-37, were synthesized as previously described [16] and isolated as described above.

### Circular dichroism spectrum analysis

**Fig. 1:**
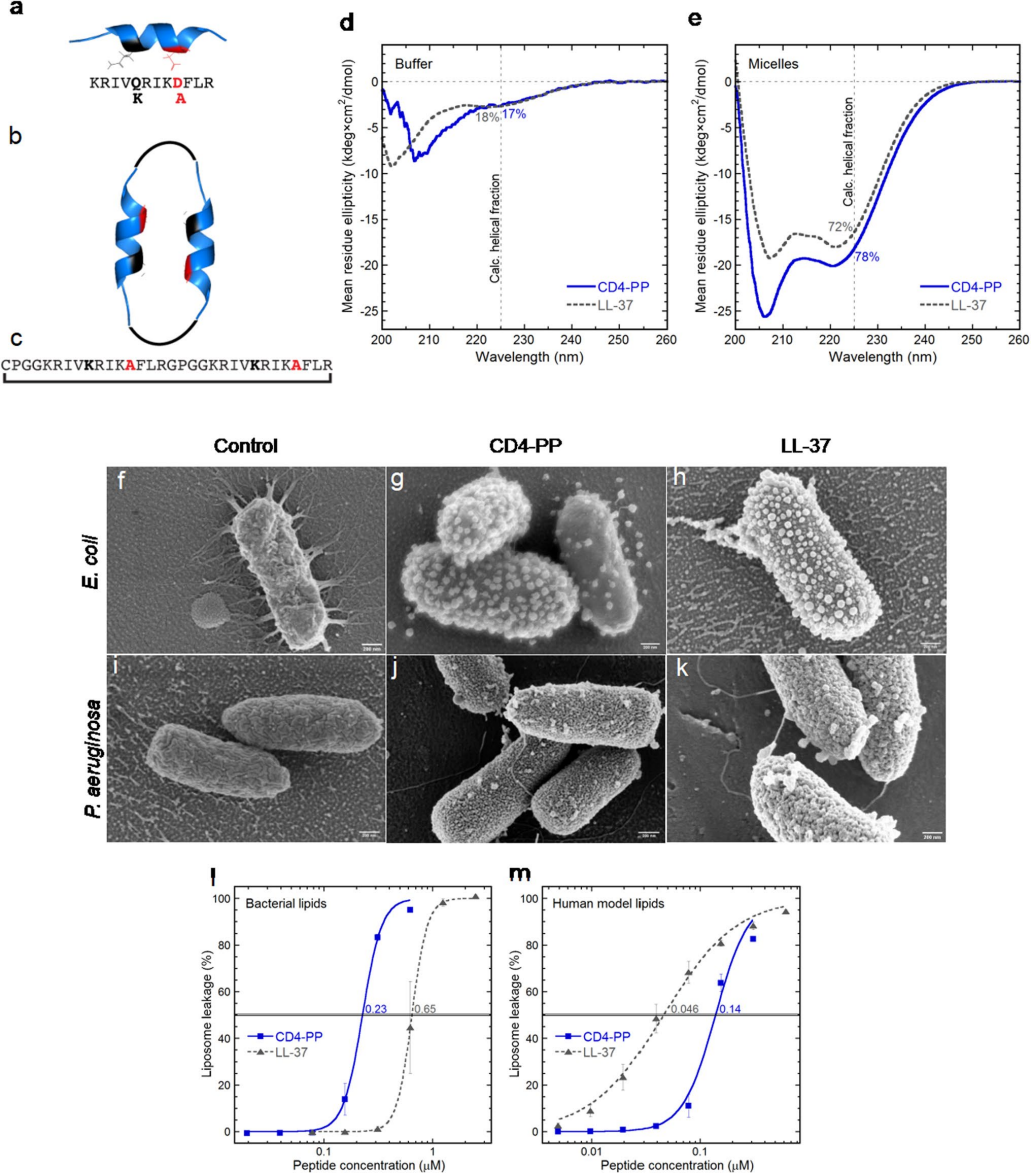
The design, structure, and mechanism of action of CD4-PP. The three-dimensional structure of the minimum active region of LL-37, known as KR-12, which was optimized with amino acid substitutions Q5K, D9A (**a**), and the structure of CD4-PP (**b**) along with the amino acid sequence (**c**). The circular dichroism (CD) spectra of LL-37, and CD4-PP in the presence or absence of lyso-PG micelle buffer (**d** and **e**, respectively). The background and baseline corrected data represent 10 µM peptide in 1 mM Tris buffer (pH 7.4, 37 °C). These micelles (present at 1:1 peptide-to-micelle ratio) represent the negatively charged microbial membrane environment, having the predominant anionic lipid headgroup and hydrophobic core thickness similar to that of Gram-negative bacterial membranes. The mean residue ellipticity is plotted against the wavelength. The relative percentage of α-helical content is determined from the signal intensity at 225 nm using a poly-Lys reference. In Tris buffer, the peptides form random-coils, whereas in micelles the peptides adopt a predominately α-helical conformation. Representative scanning electron microscopy images of *E. coli* CFT073 (**f**) and *P. aeruginosa* (ATCC 27853; **i**) exposed to CD4-PP and LL-37 as a reference, for 1 h at 37 °C. CD4-PP induced extensive blebbing in *E. coli* (**g**). A marked increase in surface roughness and few blebs were observed in CD4-PP-treated *P. aeruginosa* (**j**). The changes in bacterial morphology caused by LL-37 on *E. coli* (**h**) and *P. aeruginosa* (**k**) were similar to the ones observed after CD4-PP exposure, (**g**) and (**j**), respectively. The property of inducing membrane permeabilization was assayed through liposome leakage by measuring the level of carboxyfluorescein efflux after 45 min of incubation at 37 °C. Liposomes were made from *E. coli* polar lipid extract (**l**) or POPC:cholesterol (3:2 molar ratio; **m**), the latter representing a simplified human cytoplasmic model. Compared to LL-37, CD4-PP induced leakage at lower concentrations on bacterial liposomes and higher concentrations on the human model

The circular dichroism (CD) spectrum was assessed using methods as previously described [17]. The α-helical contribution to the secondary structure of the peptides was determined using a JASCO J810 spectropolarimeter (JASCO Corporation) monitoring changes in the 200–260 nm range at 37 °C in 10 mM Tris–HCl buffer (pH 7.4) with stirring in a 1 cm quartz cuvette. Signals from the peptides, at a concentration of 10 µM, were measured in buffer alone and with 1 mM 16:0 lyso-PG (1-palmitoyl-2-hydroxy-sn-glycero-3-phospho-(1′-rac-glycerol)) sodium salt by Avanti Polar Lipids (equating to an approximate 1:1 peptide-to-micelle ratio). Each spectrum depicted in Fig. 1d, e is the mean from 10 accumulations at a rate of 50 nm/min. The solvent background contribution was subtracted for each wavelength as well as the baseline drift for each measurement (normalized at 260 nm, where no peptide signal is present). The quantification of α-helix composition was calculated at 225 nm and compared to a poly-l-Lys reference (30–70 kDa) from Sigma-Aldrich) in 0.1 M NaOH (100% helix) and 0.1 M HCl (100% coil).

### Nuclear magnetic resonance (NMR) experiments

Freeze-dried peptides (~ 1 mg) were dissolved in 220 μL of H2O/D2O (9:1, v/v) at pH ~ 5 for NMR analysis. Spectra were acquired with and without the addition of either deuterated  sodium dodecyl sulfate (SDS; Merck; peptide:SDS 1:40 molar ratio) or lyso-phosphatidylglycerol (lyso-PG) micelles 1 mM, 16:0 lyso-PG (1-palmitoyl-2-hydroxy-sn-glycero-3-phospho-(1’-rac-glycerol)) sodium salt by Avanti Polar Lipids. One- and two-dimensional spectra (^1^H–^1^H TOCSY, ^1^H–^1^H NOESY) were acquired and processed as previously described [17]. NMR spectra were acquired on a Bruker Avance Noes 600 MHz TCI (CRPHe TR-^1^H &^19^F/^13^C/^15^ N 5 mm-EZ) spectrometer.

### *E. coli* liposome leakage assay

The liposome leakage assay was performed as described previously [20], here using either *E. coli* polar lipid extract or 1-palmitoyl-2-oleoyl-sn-glycero-3-phosphocholine (POPC, both from Avanti Polar Lipids) and cholesterol (Sigma-Aldrich). In short, lipid bilayers were deposited on round-bottom flask walls and re-suspended at 55 ºC in Tris buffer containing of 100 mM 5(6)-carboxyfluorescein. Due to shorter interlamellar distance together with a higher resistance to undulations, the zwitterionic, cholesterol-containing composition, POPC/cho, (60/40 mol%), require repeated freeze–thawing. Multilamellar structures and polydispersity were reduced by repeated extrusion through 100-nm polycarbonate membranes and un-trapped carboxyfluorescein was removed by gel separation. Membrane permeability was measured by monitoring carboxyfluorescein efflux from the liposomes to the external low concentration environment, resulting in loss of self-quenching and an increased fluorescence signal. The leakage experiments were performed on a 96-well plate format. Wells were prepared with a two-fold serial dilution of the peptides in Tris buffer. The plates were pre-heated to incubation temperature (37 °C) and liposomes subsequently administered (to a final lipid concentration of 10 μM in 200 µl) by automated dispenser. The effect on liposome permeability for each peptide concentration was monitored for 45 min, at which point leakage had largely subsided. Results shown represent the mean from triplicate experiments with standard deviations and are presented as percent of total leakage generated with Triton X-100 and subtraction of the baseline value. The EC_50_ values are (when applicable) calculated using sigmoidal dose response curves, with the leakage percentage (0–100 constraints) as a function of the peptide concentration (log10).

### Bacterial strains and cultures

The following uropathogenic bacterial species were used for the in vitro experiments, *E. coli* CFT073; *E. coli* ESBL-producing (CCUG 55197); *K. pneumoniae* (ATCC 25955); *K. pneumoniae* multidrug resistant (MDR) (CCUG 58547); and *P. aeruginosa* (ATCC 27853). Clinical isolates were obtained from the Department of Clinical Microbiology, Karolinska University Hospital, Solna, Sweden. Bacteria were cultured aerobically overnight on blood agar plates at 37 °C. For each bacterial species, 20 clinical isolates were included. For further assessment of biofilm formation, the *E. coli* isolate #12 from a child with pyelonephritis was chosen. The isolate expresses both curli and cellulose, as well as type 1 fimbriae and produces biofilm as measured on microtiter plates One-step knockout of *bcsA* and *csgBA* were carried out using the methods as described previously [21]. The following mutants were used: WE1 *bcsA*::Cm (deficient in cellulose production), WE11 *csgBA*::Cm (deficient in curli production), and WE16 *csgBA bscA*::Cm (deficient in both cellulose and curli production). *E. coli* CFT073 was used for all experiments where nothing else is stated.

### Minimum inhibitory concentration (MIC) assay

MIC of CD4-PP was evaluated against the type strains and the 20 clinical bacterial isolates using a two-step micro-dilution assay adapted for testing AMPs [22]. The concentrations of CD4-PP were prepared double strength with two-fold serial dilutions ranging from 50 to 0.78 µM in 50 µL of 10 mM Tris buffer in a U-bottom 96-well plate (Corning Incorporated).

Bacteria were grown to mid-log phase before diluting to a final concentration of 1 × 10^6^ CFU/mL in 10 mM Tris buffer using a Densichek Plus (BioMérieux). To each well, 50 µL suspension was added and incubated at 37 °C for 1 h and the final concentrations of peptide tested were from 25 to 0.39 µM. Afterwards, 5 µL of 20% (w/v) tryptic soy broth (TSB) was added and incubated at 37 °C for another 16–18 h, after which the MIC was measured. The MIC was read spectrophotometrically at 595 nm. MIC was defined as the lowest concentration of CD4-PP which inhibits bacterial growth. The effects of salt ions on the antibacterial activities of CD4-PP were assessed against *E. coli* (ATCC 25922) and *P. aeruginosa* (ATCC 27853) type strains. The following salts were used: sodium chloride (NaCl), ammonium chloride (NH_4_Cl), calcium chloride (CaCl_2_), magnesium chloride (MgCl_2_) and ferric chloride (FeCl_3_). All salts were purchased from Merck. The final concentrations of salts added to the aforementioned MIC assays were 150 mM NaCl, 6 μM NH_4_Cl, 2.5 mM CaCl_2_, 1 mM MgCl_2_, and 4 μM FeCl_3_.

### Biofilm formation

To investigate if CD4-PP can prevent the formation of bacterial biofilm, the crystal violet assay was used. Based on a dose response titration, 10 µM of CD4-PP was the optimal concentration. In brief, 50 µL of 10^6^ CFU/mL of uropathogenic *E. coli* CFT073 in 150 µL LB broth without salt with or without CD4-PP were added [23]. After 3 days of incubation at 37 °C, the old media was discarded, and the wells were washed 3 × with sterile water. Each well was stained for 15 min with 0.3% crystal violet. Non-bound crystal violet was removed, and the wells were washed 3 × with PBS. Bound crystal violet was dissolved using a EtOH:acetonitrile solution (4:1, v/v) and the absorbance was read at 570 nm. For dissolving mature biofilm, bacteria were grown as described previously for 48 h without the addition of peptide. After 48 h, old media was discarded and replaced with 200 µL of LB media with or without 20 µM of CD4-PP and incubated for another 24 h. Biofilms were washed and stained as described previously.

For measuring the formation of biofilm by *P. aeruginosa* at early timepoints, the above protocol was used, but the washing was performed after 30 min, 1 h, 2 h, and 4 h after inoculation.

### Scanning electron microscopy

Scanning electron microscopy (SEM) was used to evaluate bacterial morphology after treatment with CD4-PP. SEM was performed based on the methods described previously [24]. *E. coli *CFT073 and *P. aeruginosa (*ATCC 27853) were grown to mid-log phase and thereafter diluted in 10 mM Tris buffer at a cell density of 10^8^ CFU/mL. Bacterial suspensions (100 µL) were then incubated with CD4-PP (final concentration 3.9 µM and 7.8 µM for *E. coli* and *P. aeruginosa*, respectively) for 1 h at 37 °C. The peptide concentration corresponded to the MIC found for the high cell density condition (10^8^ CFU/mL) in the case of *E. coli* and to 5 times the MIC at standard cell density in the case of *P. aeruginosa*. Untreated bacteria served as control and bacteria exposed to LL-37 (MIC corresponding to 15.62 μM for both *E. coli* and *P. aeruginosa*) were used as reference. After the exposure experiment, bacterial suspensions (100 µL) were deposited on Nunc™ Thermanox™ coverslips (Thermofisher Scientific) and left to adhere for 1 h. Bacterial cells were then fixated with 2.5% glutaraldehyde (VWR Chemicals, USA) in PBS overnight at 4 °C, washed 2 × with PBS and deionized water, post-fixated with 1% osmium tetroxide (Sigma-Aldrich, USA) for 1 h (with the exception of the *E. coli* control) and washed with PBS and deionized water. The samples were then dehydrated with a series of ethanol concentrations (10, 30, 50, 70, 90 and 100% (v/v)) followed by further dehydration with hexamethyldisilazane (HMDS; Sigma-Aldrich) solutions (HMDS:ethanol 1:2, 2:1 and 100% HMDS). HMDS solution was removed, and samples were left to air dry overnight.

Coverslips were mounted on carbon stubs and sputter-coated with a conductive thin layer of gold and palladium. Bacterial cells were imaged using a LEO 1550 SEM instrument (Zeiss) with an InLens detector at 2–3 kV acceleration voltage and at 2–3 nm working distance.

### Cell lines and culture conditions

Human uroepithelial cells, 5637 (HTB-9, American Type Culture Collection) and T24 (HTB-4, American Type Culture Collection) were cultured in RPMI 1640 and McCoy’s 5a medium (Life Technologies), respectively, and supplemented with 10% heat inactivated fetal bovine serum (FBS; Life Technologies). All cells were incubated at 37 °C with 5% CO_2_ and 80% humidity.

### Microtiter plate method to measure cytotoxicity of CD4-PP on uroepithelial cells

The effect of varying concentrations CD4-PP (100 µM to 0 µM, using twofold dilutions) on T24 and 5637’s metabolic activity was determined using an XTT assay using methods described previously [25]. At 50 µM of CD4-PP, a cytotoxic effect was observed.

### Cell infection assays

5637 and T24 uroepithelial cells were seeded at ~ 80% confluency in 24-well plates (Costar). Two experimental conditions were used: either treatment with 2 µM of CD4-PP together with bacterial infection or 2 h after initial infection. These conditions were compared to untreated controls. During experiments, the media did not contain FBS.

Approximately, 80% confluent 5637, and T24 cells were infected with a multiplicity of infection (MOI) 5 of *E. coli* CFT073, ESBL-producing *E. coli* (CCUG 55971), *K. pneumoniae* (ATCC 25955), MDR *K. pneumoniae* (CCUG 58547), or *P. aeruginosa* (ATCC 27853)*.* After infection, cells were incubated at 37 °C at 5% CO_2_ and 80% humidity.

After 2 h of infection, old media and non-adherent bacteria were removed by washing the wells 3 × with 500 µL of PBS and fresh media, with or without CD4-PP, was added. Cells were re-incubated for another 2 h at 37 °C with 5% CO_2_.

After 2 h (total of 4 h) of infection, old media was removed, and cells were washed. To collect cell-associated and intracellular bacteria, cells were lysed with 200 µL of ice-cold 0.1% Triton X-100 in PBS and scraped thoroughly. Lysates were serially diluted in PBS and plated on blood agar plates and viable count was performed.

### Total RNA extraction and real-time PCR analysis

T24 and 5637 uroepithelial cells were seeded in 24-well plates and four conditions were established; cells treated with or without 10 µM of CD4-PP and with or without infection with MOI 5 of *E. coli* CFT073*.* Cells were either treated and/or infected for 2 h before RNA isolation.

RNA extraction, cDNA preparation and qPCR were performed as described previously [26]. Gene targets used in this study included: human beta-actin (*ACTB*), IL-8 (*CXCL8*), occludin (*OCLN*), claudin-14 (*CLDN14*), LL-37 (*CAMP*), HBD2 (*DEFB4A*), RNase7 (*RNASE7*), and psoriasin (*S1007A*). Primer sequences are shown in Supplementary Table S3.

### Immunofluorescence analysis

Uroepithelial cells, 5637, were grown on glass coverslips (VWR) in a 24-well plate containing RPMI-1640 with 10% FBS until they reached 80–90% confluence. Cells were pre-treated for 4 h with or without 10 µM CD4-PP before infection for 2 h with MOI 5 of *E. coli* CFT073. Cells were fixed and stained using methods as previously described [27] and processed for immuno-staining with anti LL-37 (Santa Cruz Biotechnology), anti-claudin-14 (Abcam), and anti-occludin (Abcam) at 1:200 dilutions, followed by respective Alexa Fluor 647, 488, and 594 conjugated secondary antibody (Life Technologies) at 1:400 dilution. Cells were counter-stained using 2.5 µg/mL 4′,6-diamidino-2-phenylindole (DAPI; Invitrogen).

Imaging was performed using 63X oil immersion objective of Zeiss LSM700 confocal microscope (Carl Zeiss). Fluorescence intensity and area of each cell were quantified by manually defining the boundaries around each cell with the FIJI software [28]. For each cell, the intensity/area ratio was calculated. Since each view-field contained 5–15 cells, the average of intensity/area values of all cells in the view-field was calculated. Expression of the target value was then normalized to corresponding control average value, making control as 1. At least three view-fields per slide each were quantified from three independent experiments.

### Enzyme-linked immunosorbent assay

Cell-free cultures from in vitro experiments were collected using standard protocol [29]. IL-8 was analyzed according to the manufacturer’s recommendations (R&D Biosystems, USA).

### Prevention of *E. coli* adhesion to urinary catheters

The ability of CD4-PP to limit the adhesion of uropathogenic *E. coli* to urinary catheters was assessed using the methods described in Piktel et al. (2021) with some modifications [30]. In brief, a commercially available urinary catheter (Dentsply) was cut into ~ 1 cm long pieces under sterile conditions. The catheters are hydrophilic polyolefin-based elastomer coated with polyvinylpyrrolidone, which when hydrated form a hydrogel. The saline packet accompanying each catheter was split into two portions: one containing 10 µM of CD4-PP, and one without. Catheter pieces were equally distributed between the two conditions, thoroughly mixed and stored overnight in solution at 4 °C. Culture negative human urine samples were collected at the Karolinska University Hospital and stored at 4 °C until use. Individual catheter pieces were placed in each well in a 24-well plate flooded with urine. Each well was then spiked with 10^6^ CFU/mL of *E. coli* CFT073 and incubated at 37 °C with 5% CO_2_ and 80% humidity without shaking. The culture negative urine was replaced every 24 h per sample. After 24 h, 48 h, and 72 h, catheter pieces were transferred to individual 10 mL tubes, washed with PBS to remove non-adherent bacteria. After washing, 1 mL of PBS was added to each tube and kept on ice. Catheter pieces were sonicated for 5 min at 40 kHz to detach adhered bacteria and viable count was performed. Samples without the synthetic peptide served as controls and remaining samples were normalized to the controls. After 24 h, the urine was serial diluted and viable counts were performed.

### Statistics

All statistical tests were performed in GraphPad Prism, version 9.2.0. Statistical outliers, as defined by Grubb’s test, were excluded from the datasets. Statistical comparisons between two variables were performed by paired t-test where appropriate, while those involving multiple comparisons were done by one-way ANOVA. Differences with p-values less than 0.05 were regarded as being statistically significant.

## Results

### Design and synthesis of CD4-PP

The choice of KR-12 (Q5K, D9A) as a starting point in the design of CD4-PP was based on our previous work, which showed the substitutions at positions 5 and 9 to be crucial to improve antimicrobial activity [16]. The design was then completed by coupling two KR-12 (Q5K, D9A) monomers into a seamless peptide backbone via linkers composed of four amino acid residues. Pro residues were introduced in the linker regions with the purpose to induce a helix–turn–helix–turn motif in the mature peptide, and hence to keep the structure of the two adjoining α-helical monomers. The design of CD4-PP and amino acid sequence is detailed in Fig. 1a–c.

CD4-PP was assembled as a linear chain including a C-terminal diaminobenzoic acid (Dbz) group and a N-terminus Cys. The Dbz group was converted into an *N*-benzymidazolinone (Nbz) group to facilitate peptide backbone cyclization by native chemical ligation [19, 31]. Peptide identity was confirmed using LC–MS, and purity determined using RP-HPLC (at 215 nm) to > 95% (see Supplementary Fig. S1). Expected and observed masses, together with their calculated net charge and hydrophobicity, are shown in Supplementary Table S1.

### CD4-PP adopts an α-helical conformation in membrane mimetic environments

To investigate if the helical structure of the monomers is maintained in CD4-PP, it was first analyzed using NMR in solution. Spectra showed limited dispersion and broadened peaks demonstrating that CD4-PP is unstructured in water. SDS was then added, to determine if structure changes in environments mimicking membranes. Since the complexity of the NMR spectra made the assignments impossible, the exact structures under such conditions were unknown. To improve similarity to lipid membranes, NMR was performed with lyso-phosphatidylglycerol (PG) micelles added. These micelles are intended to mimic the anionic microbial membrane composition with a micelle diameter equivalent to biological membrane thickness and used at a 1:1 peptide-to-micelle ratio. Combined with great overlap, the high concentration of PG made the spectra not possible to assign (Supplementary Fig. S2).

In contrast to NMR, analysis via circular dichroism (CD) spectroscopy revealed secondary structure characteristics. CD spectra of the peptides were recorded in Tris buffer with or without PG micelles. Both CD4-PP and LL-37 adopted random coil confirmations, which are analogous with the NMR experiments above (Fig. 1d). However, in Tris buffer containing micelles, the CD spectra of CD4-PP exhibited distinct α-helical profile (78% α-helical content), which was greater than that of LL-37 (72% α-helical content, Fig. 1e).

### CD4-PP has enhanced proteolytic stability

A major limitation of AMPs as potential drug candidates is due to their inherent proteolytic instability, and endogenous AMPs are readily degraded by both host- and pathogen-proteases [14]. It is known that the parent peptide, LL-37, is cleaved by the bacterial protease aureolysin [16]. Therefore, the stability of CD4-PP was tested against this enzyme. The percentage of the intact peptide at different time points was monitored using LC–MS, and compared to LL-37 as a control. LL-37 is rapidly degraded within minutes. In contrast, CD4-PP is stable for up to 6 h (Supplementary Fig. S2).

### CD4-PP has antibacterial activity against pathogenic bacteria

To investigate the antimicrobial activity of the synthetic peptide CD4-PP, we performed minimum inhibitory concentration (MIC) assays for uropathogenic species in concentrations below cellular cytotoxicity. CD4-PP was active against both clinical and type strains (*n* = 20 and *n* = 2, respectively, of each tested species) in low concentrations between 0.78 and 1.56 µM. The MIC for *E. coli* CFT073 and the ESBL strain (CCUG 55971) were 0.78 µM. *Klebsiella pneumoniae* (ATCC 25955), MDR *K. pneumoniae* (CCUG 58547), *Pseudomonas aeruginosa* (ATCC 27853) had MICs of 1.56 µM. All clinical isolates had either the same or lower MICs. Increasing salt concentrations are well known to affect the antimicrobial activity of AMPs [32]. When challenging *E. coli* (ATCC 25922) and *P. aeruginosa* (ATCC 27853) with CD4-PP under varying salt concentrations, we found that the addition of NaCl and CaCl_2_ increased the MIC of LL-37, but none of the salts affected the MIC of CD4-PP (Supplementary Table S2).

### Membrane deformation caused by CD4-PP

To visualize the effect of CD4-PP on the morphology of *E. coli* CFT073 and *P. aeruginosa* (ATCC 27853), scanning electron microscopy (SEM) was used. Bacteria were imaged after 1 h of exposure to the peptide at its MIC. A marked effect of the peptide on the membrane of *E. coli* was evident when comparing the morphology to the untreated control (Fig. 1f). The peptide caused formation of blebs all over the bacterial surface, with cell debris in the form of small spheres observed near or on the surface of the individual cells, most probably corresponding to detached blebs (Fig. 1g). *E. coli* CFT073 exposed to CD4-PP was comparable to the morphology observed in LL-37 exposed bacteria, used as an internal control (Fig. 1h), indicating a similar peptide–membrane interaction between CD4-PP and LL-37. Blebs were also observed in the CD4-PP treated *P. aeruginosa* (Fig. 1j)*,* but in this case, fewer blebs per cell were observed as compared with *E. coli*. Moreover, peptide-treated *P. aeruginosa* showed a marked increase in the roughness of the surface membrane compared with untreated bacteria with no significant differences between CD4-PP and LL-37.

### CD4-PP shows higher activity towards bacterial mimicking membranes in liposome models

Based on the SEM imaging showing membrane damage, the potential for membrane permeabilization was studied using a liposome leakage assay on model membranes. This was accomplished by measuring the efflux of carboxyfluorescein from liposomes of either *E. coli* polar lipid extract or the human model lipid system POPC:cholesterol. The parent peptide LL-37 has a well-documented antimicrobial activity based primarily on membrane disruption and was used as a reference. Results show that CD4-PP induced substantial leakage from *E. coli* liposomes with an EC_50_ threefold lower than LL-37 (Fig. 1l). In terms of peptide induced permeability on the human model membrane system, (Fig. 1m), the situation was reversed, with LL-37 instead being more potent and having a threefold higher activity than CD4-PP. Liposome leakage for CD4-PP strongly indicated that the peptide is membrane disruptive and is more targeted towards bacterial membranes as compared with LL-37. The homogenous alkyl groups of synthetic POPC-based liposomes seem to render them more susceptible to peptide-generated leakage, likely due to the inability of such homogenous lipid compositions to accommodate membrane curvature stress by demixing [20]. Therefore, we refrain from comparing results from the two liposome systems in absolute terms and instead focus on the relative results from within each system.

### CD4-PP prevents new biofilm and can dissolve established biofilm

**Fig. 2:**
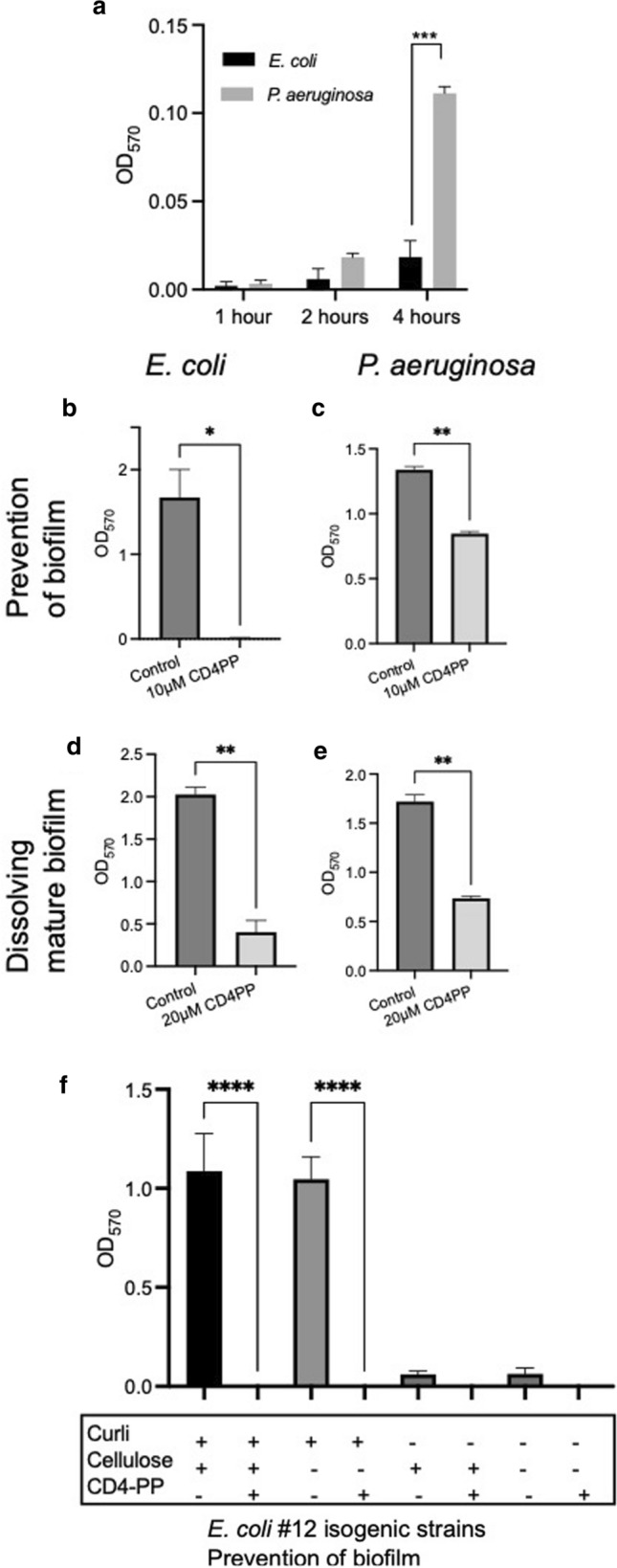
Biofilm formation occurs early and CD4-PP prevents the formation of new biofilm and dissolves mature biofilm. The early formations of biofilm were detectable in *E. coli* CFT073 and *P. aeruginosa* (ATCC 27853) 2 and 4 h after seeding, with *P. aeruginosa* producing significantly more biofilm as compared with *E. coli* (**a**)*.* 10 µM CD4-PP treatment was able to significantly prevent the formation of biofilm by *E. coli* and *P. aeruginosa* (**b** and **d**), and 20 µM of CD4-PP was capable of dissolving mature biofilm (**c** and **e**). Using the *E. coli* #12 isogenic strains, a significant reduction of biofilm was observed in curli positive strains, but not for curli deficient strains (**f**)

Uropathogens, such as *E. coli* and *P. aeruginosa*, utilize a variety of different strategies to evade clearing by the immune responses. These include the expression of several extracellular compounds involved in surface adhesion, cell–cell interaction, and production of matrix. In conjunction, these factors are involved in the production of biofilm, which shields bacteria from external threats. Formation of biofilm is considered to start early after bacteria have adhered. Interestingly, we here show that *P. aeruginosa* (ATCC 27853) biofilm formation is initiated earlier and has significantly more biofilm after 4 h compared *E. coli* CFT073 at the same time point (Fig. 2a, *p*  < 0.001). Moreover, we observed that 10 µM CD4-PP significantly prevented the formation of new biofilm produced by *E. coli* CFT073 and *P. aeruginosa* after 72 h (Fig. 2b and c *p* < 0.05, and *p* < 0.01, respectively). In addition, 20 µM CD4-PP was able to significantly dissolve mature *E. coli* and *P. aeruginosa* biofilms (Fig. 2d and e *p* < 0.01, and p < 0.01, respectively).

Uropathogenic *E. coli* isolates typically express several extracellular compounds, such as amyloid fiber curli, and cellulose, which together produce a robust biofilm [33]. To investigate to what extent these extracellular compounds are affected by CD4-PP, we utilized isogenic mutants, deficient of cellulose and/or curli (WE1 *bcsA*::CM and WE11 *csgBA*::Cm, respectively) [21]. Biofilms formed by the wild-type *E coli #*12 and the isogenic mutant deficient of cellulose but carrying curli (#12 wild-type and #12∆Cellulose, WE1 *bcsA*::CM) were significantly inhibited by 10 µM of CD4-PP treatment, indicating that curli was the target (Fig. 2f).

### CD4-PP has immunomodulatory effects in uninfected uroepithelial cells

It is well known that uroepithelial cells produce AMPs after stimulation by metabolites and during infection [27, 34]. Therefore, we speculated that uroepithelial cells may secrete AMPs not only during infection, but also in response to CD4-PP.

**Fig. 3:**
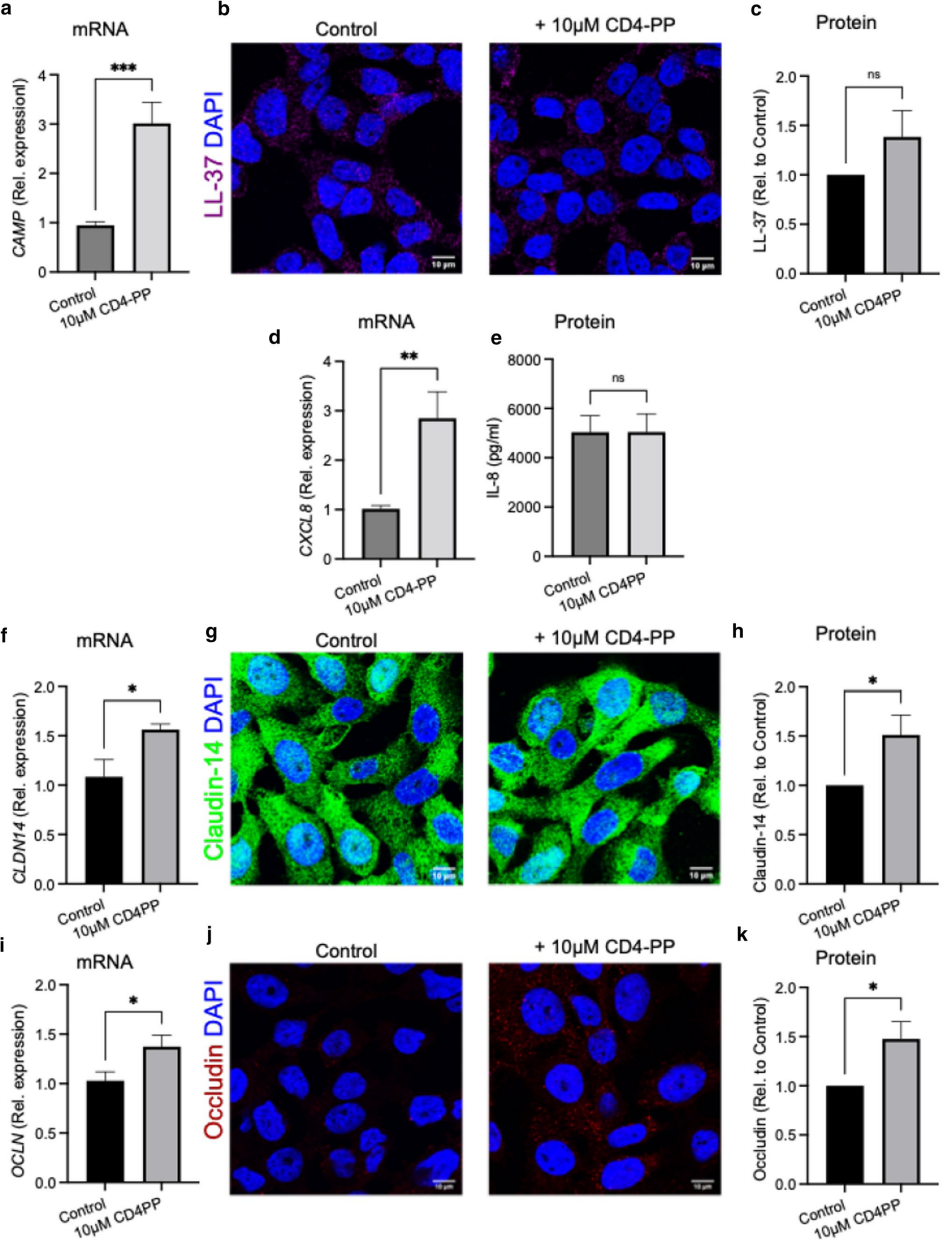
CD4-PP has immunomodulatory effects in vitro in uninfected uroepithelial cells. Uninfected 5637 human urothelial cells, stimulated with CD4-PP, demonstrate mRNA expression of *CAMP* (**a**) and representative images (**b**) depicting LL-37 stained with Alexa Fluor 647 (magenta), nucleus stained by DAPI for nucleus (blue) Relative average intensity of LL-37 (**c**). Uninfected T24 uroepithelial cells, demonstrating mRNA expression of *CXCL8* (**d**) and protein level for IL-8 (**e**). mRNA expression of *CLDN14* as measured using RT-PCR (**f**). Representative images (**g**) depicting claudin-14 stained with Alexa Fluor 488 (green), nucleus stained by DAPI for nucleus (blue). Relative average intensity of claudin-14 (**h**). mRNA expression of *OCLN* as measured using RT-PCR (**i**). Representative images (**j**) depicting occludin stained with Alexa Fluor 594 (red), nucleus stained by DAPI for nucleus (blue). Relative average intensity of occludin (**k**). Relative mRNA expression of targeted genes was performed in three independent sets in triplicate. All microscopy imaging and densitometry analysis were performed in three independent experiments, with each experiment consisting of 4–5 random view-fields. Statistical analysis of densitometry was paired *t* tests. All experiments were performed in human uroepithelial cells, 5637 and T24, and were treated for either 2 h or 4 h with 10 µM CD4-PP for mRNA and protein experiments, respectively. Treatment was compared to untreated controls. Significance levels *P < 0.05; **P < 0.01; ***P < 0.001

Stimulation of the uroepithelial cell line, 5637, with 10 µM CD4-PP significantly increased the expression of the parent antimicrobial peptide LL-37 on the mRNA level, measuring the gene *CAMP* (Fig. 3a). On the protein level, treatment itself did not increase the expression of LL-37 in uninfected conditions (Fig. 3b and c) as measured by immunofluorescence. Although CD4-PP increased the expression of *CAMP*, it did not affect the gene expression of other investigated AMPs: *DEFB4A, RNASE7* or *S100A7* (Supplementary Fig. S3a–c).

Uroepithelial cells, T24, treated with 10 µM CD4-PP showed an upregulation of *CXCL8*, encoding for the neutrophil chemokine attractant IL-8 (Fig. 3d) as compared with untreated cells. However, no differences were observed of the secreted IL-8 protein (Fig. 3e). It is well known that tight junction proteins contribute to forming a protective barrier maintaining tissue impermeability [35]. Treatment of uninfected uroepithelial cells with CD4-PP increased the expression of the tight junction proteins claudin-14 and occludin on both the mRNA and protein levels (Fig. 3f–k).

### Immunomodulatory effects of CD4-PP during infection

**Fig. 4:**
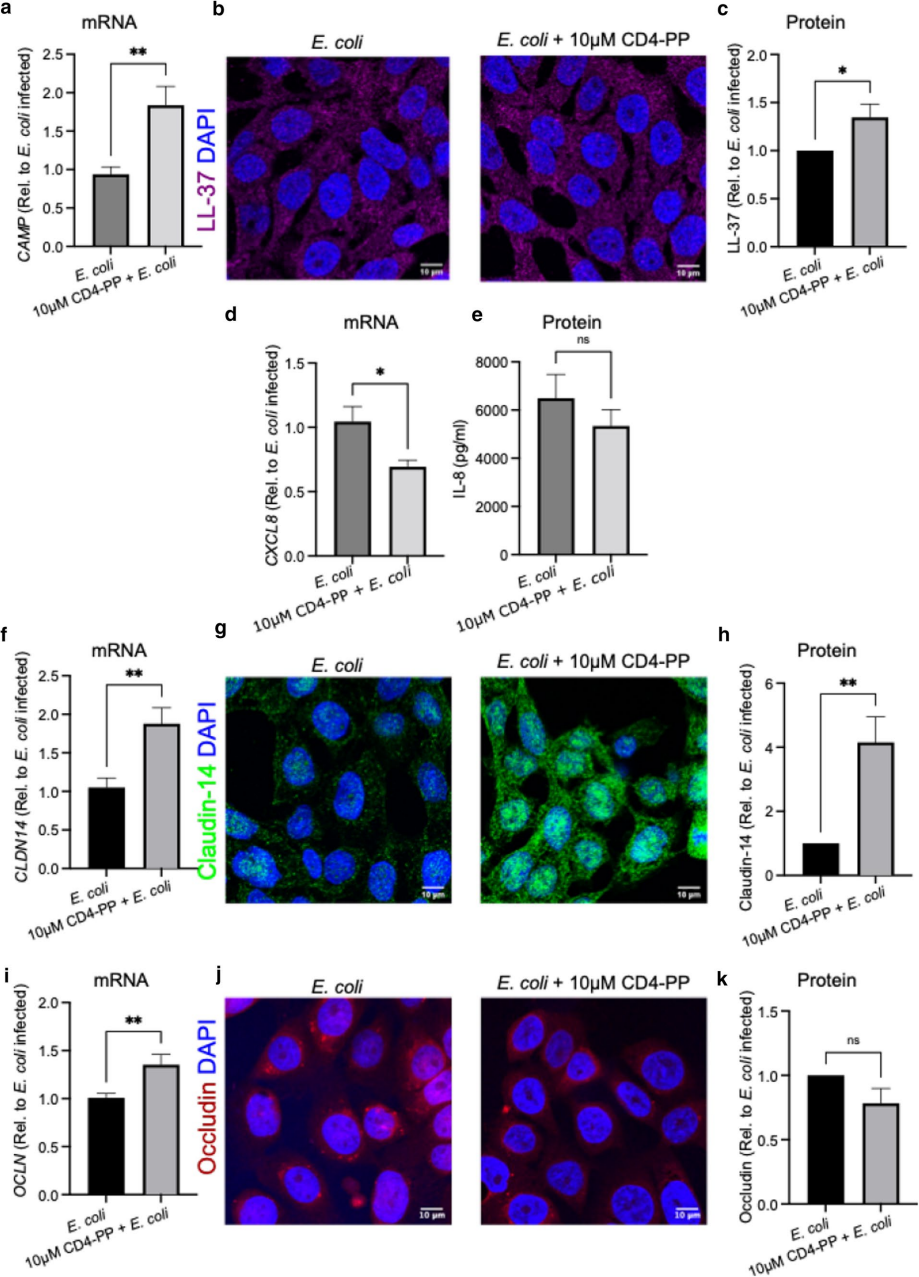
During *E. coli* infection, CD4-PP has immunomodulatory effects. Human urothelial cells, 5637, were infected with *E. coli* CFT073 demonstrating mRNA expression of *CAMP* (**a**) and representative images (**b**) depicting LL-37 stained with Alexa Fluor 647 (magenta), nucleus stained by DAPI for nucleus (blue) Relative average intensity of LL-37 (**c**). T24 uroepithelial cells infected with *E. coli* CFT073, demonstrating mRNA expression of *CXCL8* (**d**) and protein level for IL-8 (**e**). mRNA expression of *CLDN14* as measured using RT-PCR (**f**). Representative images (**g**) depicting claudin-14 stained with Alexa Fluor 488 (green), nucleus stained by DAPI for nucleus (blue). Relative average intensity of claudin-14 (**h**). mRNA expression of *OCLN* as measured using RT-PCR (**i**). Representative images (**j**) depicting occludin stained with Alexa Fluor 594 (red), nucleus stained by DAPI for nucleus (blue) (**i** and **l**). Relative average intensity of occludin (**k**). Relative mRNA expression of targeted genes was performed in three independent sets in triplicate. All microscopy imaging and densitometry analysis were performed in three independent experiments, with each experiment consisting of 4–5 random view-fields. Statistical analysis of densitometry was paired *t* tests. All experiments were performed in human uroepithelial cells, 5637 and T24, and were treated for either 2 h or 4 h with 10 µM CD4-PP for mRNA and protein experiments, respectively. Treatment was compared to untreated controls. Significance levels *P < 0.05; **P < 0.01; ***P < 0.001

To ascertain the effect of CD4-PP during a potential UTI, we simultaneously treated and infected uroepithelial cells with the peptide and *E. coli* CFT073. During infection, we observed a significant upregulation of LL-37 on both the mRNA and protein levels produced by uroepithelial cells, as compared with corresponding untreated controls (Fig. 4a–c). In addition to secreting AMPs, infected uroepithelial cells release chemokines to recruit immune cells, including neutrophils and macrophages, to aid in the eradication of the infection [1]. However, during infection, the relative expression of *CXCL8* in CD4-PP-treated cells was significantly lower than the untreated cells (Fig. 4d), and no differences in secreted IL-8 protein were observed (Fig. 4e). Another important aspect to consider is the possible impact of CD4-PP on epithelial tissue integrity. As part of the innate defense strategy, infected superficial uroepithelial cells are shed to eradicate bacteria. While this is beneficial to the host, it also exposes the underlying tissue to bacterial invasion. However, during infection, only claudin-14 increased on both the mRNA and protein level, while occludin protein expression remained unchanged (Fig. 4f–k).

### CD4-PP helps protect uroepithelial cells from infection

After observing the direct antibacterial effect of CD4-PP against uropathogens and its immunomodulatory effects, we investigated whether CD4-PP would facilitate the eradication of uropathogens during in vitro infection. To mimic the natural situation, we used two-time points, treatment of 2 µM CD4-PP at the start of infection or 2 h post-infection.

**Fig. 5:**
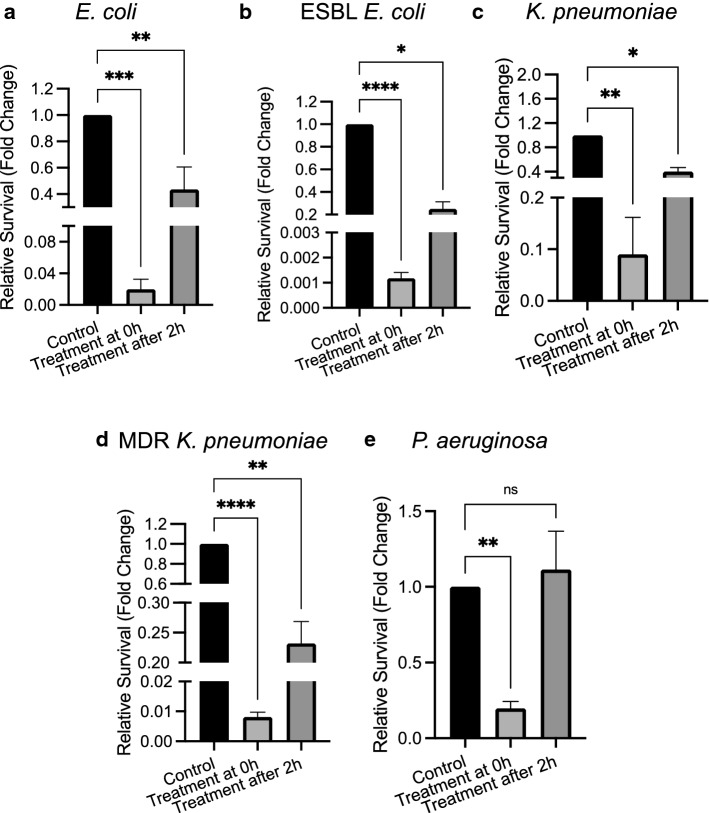
Reduced survival of uropathogens by CD4-PP treatment during in vitro infection. Survival of uropathogens *E. coli* CFT073 (**a**), ESBL-producing *E. coli* (CCUG 55971; **b**), *K. pneumoniae* (ATCC 25955; **c**), MDR *K. pneumoniae* (CCUG 58547; **d**), and *P. aeruginosa* (ATCC 27853) after infecting uroepithelial cells. Survival in treatment groups is relative to untreated controls. CD4-PP treatment was initiated at the same time as infection (0 h) or after 2 h of infection (2 h). Significance levels *P < 0.05; **P < 0.01; ***P < 0.001; ****P < 0.0001

Irrespectively of when treatment was initiated, a clear decrease in bacterial survival was observed for *E. coli* CFT073, ESBL-producing *E. coli,* non-MDR and MDR *K. pneumoniae* (Fig. 5a–d). During *P. aeruginosa* infections, only CD4-PP treatment at the time of infection reduced the survival. When the CD4-PP was added 2 h after initiation of the infection, the peptide was unable to reduce the survival of the *P. aeruginosa*-type strain (Fig. 5e).

### Potential use of CD4-PP to prevent catheter-induced UTI

**Fig. 6:**
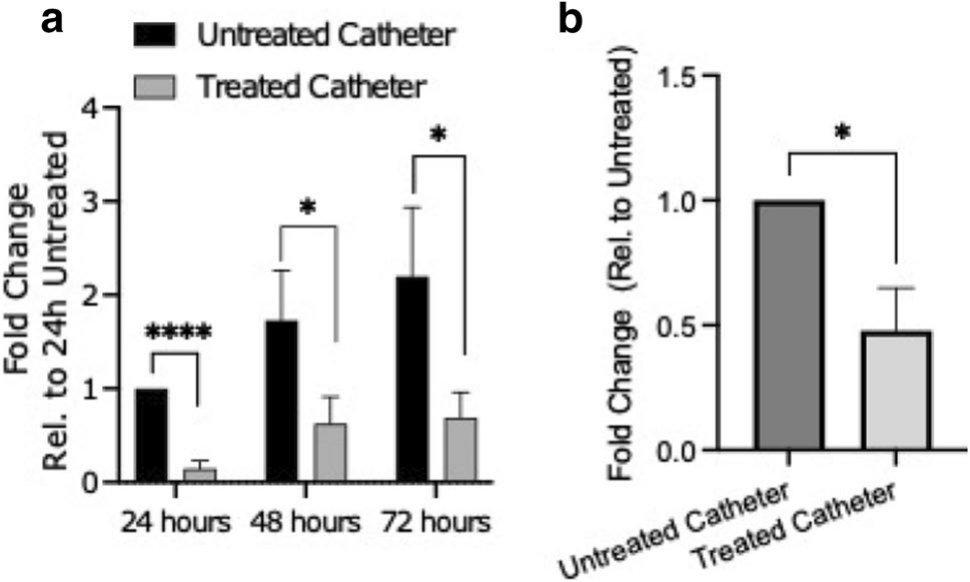
CD4-PP prevents adhesion of uropathogenic *E. coli* to catheters. The addition of 10 µM CD4-PP to the saline solution accompanying urinary catheters was able to significantly reduce the number of uropathogenic *E. coli* isolates attaching to urinary catheters after 24, 48, and 72 h (**a**). Viable counts from the urine after 24 h showed significant reduction in the number of viable *E. coli* isolates within the urine (**b**). Statistics were performed using a paired t-test. Significance levels: *P < 0.05; ****P < 0.0001

Having characterized the strong bactericidal effects of CD4-PP, we investigated whether the peptide could decrease adhesion of uropathogenic *E. coli* to urinary catheters. The formation of biofilm on urinary catheters is a common occurrence among patients and can cause discomfort and recurrent infection [36]. The material in the catheters is composed of hydrophilic polyolefin-based elastomer coated with polyvinylpyrrolidone, which when hydrated forms a hydrogel. To investigate the effect of the synthetic peptide, catheter pieces were soaked with a solution of CD4-PP. The results highlight the ability of CD4-PP to significantly limit *E. coli* attachment after 24, 48, and 72 h (Fig. 6a). In addition, CD4-PP-treated catheter pieces significantly reduced the number of uropathogenic *E. coli* in urine 24 h after inoculation (Fig. 6b, *p* < 0.05). Our results suggest potential applications of CD4-PP as a compound within storage fluids of urinary catheters.

## Discussion

Antimicrobial peptides (AMPs) present as potential alternatives to traditional antibiotics due to their broad spectrum of antimicrobial activity, slow and incomplete development of bacterial resistance, and host immunomodulatory effects. However, there are many aspects which hinder their development for clinical practice. One of the pitfalls in the development of AMPs is their inactivation or loss of antimicrobial activity by salts, serum proteins, and pH [37]. Furthermore, the typical linear AMPs, such as LL-37, are quickly inactivated through degradation by proteases released from both host and pathogens [13, 14]. Several strategies have been used to address these problems by, e.g., sequence optimization, amino acid substitution, introduction of non-peptide elements, and structural design. By developing a peptide with a cyclic backbone, we intended to increase the proteolytic stability and antimicrobial activity of the novel synthetic AMP. We observed that CD4-PP displayed enhanced stability against bacterial protease, whereas LL-37 was rapidly degraded. In addition, we demonstrated that this peptide has important advantages compared to most other AMPs: it is active at physiological salt concentrations and protects cells against infection.

CD4-PP was designed by combining two monomers, with two amino acid substitutions, of the shortest antibacterial peptide sequence found in LL-37, referred to as KR-12. These two monomers are joined together into a seamless cycle of peptide bonds, using two four-residue linker sequences. Each linker contains a proline residue to introduce a kink in the backbone intended to assist the peptide to fold into two adjacent, antiparallel α-helices. By this design, we hypothesized that cyclization would increase proteolytic stability, and by having two preformed α-helices in close proximity in their active conformation would increase activity.

To determine if the structural design was successful NMR spectroscopy was used. However, assignment of residues proved difficult because of overlapping signals and poor dispersion of peaks, which was interpreted as an indication of disordered regions. Using CD revealed secondary structure in the peptide: in buffer CD4-PP is in random coil conformation and shifts into broadly α-helical conformation in a membrane-mimicking environment. This transition was similar for LL-37. The coil-to-helix transition from solvent to membrane-mimicking environment may be interpreted as a sign of high membrane binding affinity. It is plausible that this binding also contributes to increased stability in the presence of membranes.

Differences between species were observed when evaluating the effect of CD4-PP on bacterial morphology, with *E. coli* showing more bleb formation than *P. aeruginosa.* However, no pores were observed in *E. coli* or *P. aeruginosa* treated with CD4-PP or LL-37. Interactions of Gram-negative bacteria with cationic AMPs not only involve components of the bacterial cell wall and lipopolysaccharide, but also cell surface receptors that vary between species and strains [21, 38, 39]. Bleb formation has been previously shown to support AMP activity in the cytoplasm of Gram-negative bacteria. Simultaneously, the production of blebs is a known stress response which can serve as a decoy for membrane active compounds [40, 41].

To assess a possible effect of bacterial membrane damage, we analyzed the induction of liposome leakage of CD4-PP. The assays clearly indicated a membrane disrupting activity for CD4-PP, similar to its parent peptide LL-37. Notably, CD4-PP displayed a six-fold higher selectivity for the bacterial target over LL-37. Moreover, CD4-PP displayed a lower EC_50_ against *E. coli* liposomes than the previous, first-generation cyclic dimers of KR-12 [17]. This first generation of peptides lack proline residues in the linker sequences, and contain native KR-12 sequences as they appear within LL-37. The massively increased membrane disruptive activity is proposed to derive from an increased membrane adoption isotherm by dimerization together with an artificially increased local concentration for each monomer (KR-12 Q5K, D9A) at the membrane, as in the case of previous analogues [17]. Overall, our results indicate that CD4-PP could be active both against the bacterial membrane and possibly against other targets within the cytoplasm.

The parent peptide LL-37 is naturally active in the urinary tract [10]. Together with encouraging antimicrobial results of CD4-PP against a small set of model organisms, we hypothesized that CD4-PP would have antibacterial activities against common urinary pathogens. Hence, CD4-PP was tested against a range of the most clinically important uropathogens. MICs were determined against clinical strains and type strains*,* including multidrug-resistant isolates: they were all in the low or sub-µM range, and far from cytotoxic to uroepithelial cells.

However, MIC assays assess the impact of a drug against pathogens in the absence of the host environment. Therefore, they may not necessarily reflect realistic treatment outcomes as they do not consider the influence of the host, which might itself be affected by the intervention [42]. To address this question, an in vitro infection model was used to determine whether the antibacterial effect remains, and if bacterial burden in infected uroepithelial cells is decreased by treatment with CD4-PP. Treatment at start of infection significantly decreased the survival of all the tested pathogenic strains. In addition, treatment given 2 h after the start of infection was successful in reducing the survival of *E. coli* and *K. pneumoniae* but was unable to clear *P. aeruginosa* infection.

UTIs are associated with the formation of biofilm [43, 44]. It is well known that biofilm can harbor multiple bacterial species which can exchange genetic material, such as antibiotic resistance genes, and can complicate treatment [45, 46]. The lack of efficacy of low concentrations of CD4-PP against *P. aeruginosa* after establishment of infection could be due to early formation of biofilm. It has previously been shown that biofilms act as a diffusion membrane for antibiotics, lowering their efficacy [47]. Albeit in low quantities, the initiation of *P. aeruginosa* biofilm production was detected after 2 h. After 4 h, the difference in biofilm formation was further increased. In contrast, the earliest detectable biofilm formation by *E. coli* was detected after 4 h, in levels significantly below that of *P. aeruginosa.* This early biofilm may have contributed to the lack of efficacy of low concentrations near MIC of CD4-PP against *P. aeruginosa.* Similar results have been observed demonstrating that the combination of LL-37 engineered peptide and antibiotics were capable of disrupting *P. aeruginosa* biofilms [48]. Overall, CD4-PP is active against pathogenic species, including multidrug-resistant strains, and able to reduce the bacterial number faced by uroepithelial cells.

We also demonstrate that CD4-PP can prevent the formation of newly formed biofilm and dissolve already existing, mature, biofilm. Previous studies have shown that the addition of synthetic AMPs, including LL-37 derivatives, to medical devices significantly reduces biofilm formation [49, 50]. In addition, we have previously shown that the parent peptide, LL-37, can inhibit curli-mediated uropathogenic *E. coli* biofilm formation [21]. While eradication of uropathogens is required to prevent an infection, inhibiting the formation of biofilm during UTI is vital to further deny uropathogens the chance to establish biofilm, and potentially cause recurrent infections.

Endogenously produced LL-37 is well known to have innate immunomodulatory effects, including production of AMPs, chemokines and cytokines, in addition to its role as an antimicrobial agent [5, 6]. This prompted us to investigate whether CD4-PP would also have immunomodulatory effects. Stimulation of uroepithelial cells by CD4-PP increased production of LL-37 in infected cells on the gene and protein levels. This increase likely aids in further clearing of uropathogens by resident epithelial cells from the bladder [10]. In uninfected cells, only an increase in *CAMP* mRNA was observed. Excess LL-37 protein in uninfected cells is not necessary and might even have detrimental effects on the host due its cytotoxicity [51]. Treatment of uroepithelial cells with CD4-PP altered the expression of *CXCL8,* encoding for the pro-inflammatory neutrophil recruiter IL-8, during in vitro infection. The fact that we did not see a corresponding upregulation on the protein level could be due to CD4-PP already having killed the bacteria and upregulation of the IL-8 was therefore not necessary. In addition, it is possible that the timepoint when mRNA was harvested may not have reflected the true peak of *CXCL8* expression [52].

During UTI, uroepithelial bladder cells commonly have dysregulated tight junction proteins and infected superficial cells are shed from the bladder [53]. The clearing of bacteria harbored within infected uroepithelial cells acts as a double-edge sword as it aids in the removal of uropathogens, but exposes undifferentiated cells to invading pathogens [54]. Treatment with CD4-PP to uninfected uroepithelial cells increased the expression of the tight junction proteins claudin-14 and occludin, both on the mRNA and protein level. By increasing the expression of tight junction proteins in uninfected and infected states, CD4-PP might mitigate or even prevent further bacterial invasion through strengthening the cell-to-cell adhesion which forms a barrier against invading pathogens.

In addition to infecting cells of the urothelium, uropathogens easily attach to catheters, commonly causing health-care associated infections [55]. By adding CD4-PP to a saline solution that can accompany urinary catheters, we were successful in both reducing the attachment of *E. coli* to catheter pieces and reducing the *E. coli* burden within the urine itself. These results indicate that not only is CD4-PP active against uropathogenic *E. coli* in urine, but that the adoption of a simple strategy, such as adding CD4-PP to saline lubricating solutions, can decrease the bacterial burden and reduce the risk for infections. The continued activity of CD4-PP in human urine over several days against uropathogenic *E. coli* is noteworthy, as increased salt concentrations are known to have negative effect on the antibacterial properties of AMPs [3, 56]. Therefore, CD4-PP shows promising potential for preventing the attachment of uropathogenic *E. coli* to urinary catheters in the future. However, we observed that concentrations above the MIC of CD4-PP for *E. coli* were required to decrease the bacterial load in human urine. It is possible that physiological factors between patient urine samples had an impact on the efficacy of the peptide in urine [34, 57]. Despite this, the dual effect of the peptide on the prevention of adhesion of *E. coli* to the catheter pieces in addition to the killing effect of the peptide on planktonic bacteria within the urine should be noted.

## Conclusions

In conclusion, this work highlights the potential of a novel synthetic antimicrobial peptide as a possible therapeutic agent to prevent and treat UTIs. The peptide CD4-PP shows encouraging activity against uropathogenic bacteria, including clinical isolates and resistant strains. In particular, it has activity in an in vitro infection model, and reduces bacterial adhesion to urinary catheters, indicating a promising clinical application.

## Supplementary Information

Below is the link to the electronic supplementary material.

Supplementary file1 (DOCX 3708 KB)

## Data Availability

The datasets generated during the current study are available from the corresponding author/Karolinska Insitutet’s repository upon reasonable request.
