# Supplementary material for: A stable cyclized antimicrobial peptide derived from LL-37 with host immunomodulatory effects and activity against uropathogens
Source: Cell Mol Life Sci. 2022 Jul 11;79(8):411. doi: 10.1007/s00018-022-04440-w (PMC9276586; doi:10.1007/s00018-022-04440-w)
Supplement: Supplementary file 1 — Supplementary file1 (DOCX 3708 KB) [file 18_2022_4440_MOESM1_ESM.docx]

**
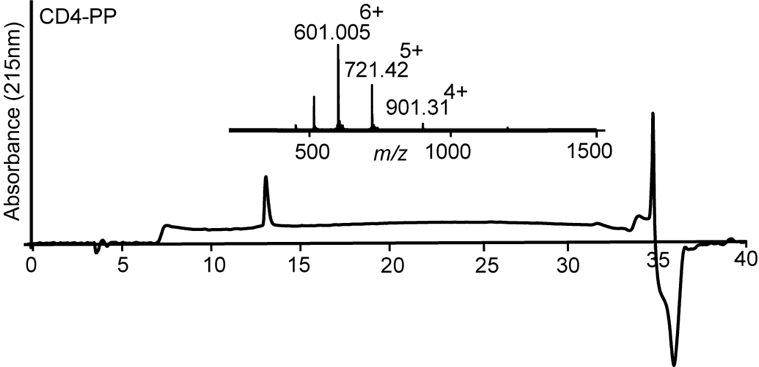
**

**
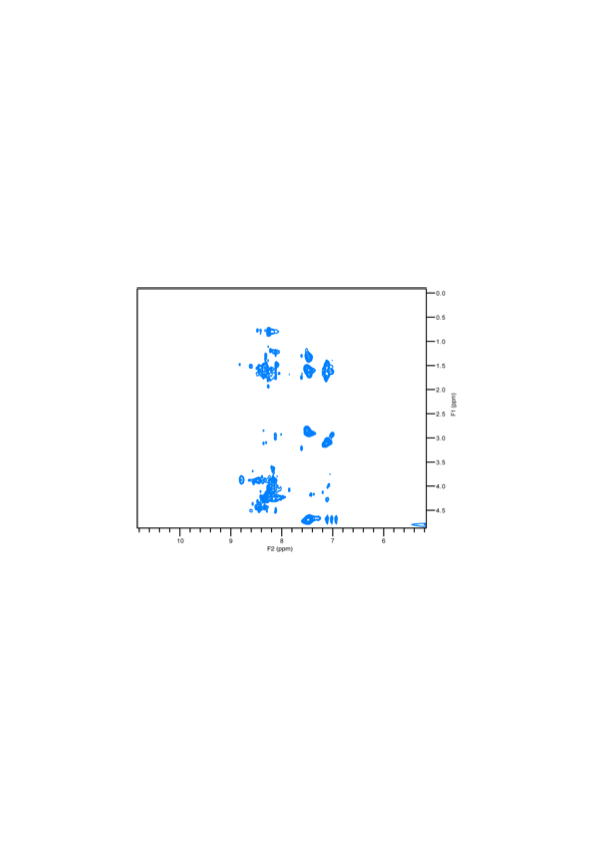
**

**Supplementary Figure S1. Physical characterization of CD4-PP.** Analytical HPLC chromatogram and LC-MS spectrum of CD4-PP TOCSY spectrum of CD4-PP displayed broadening of signals and overlapping resonances which prevented accurate sequence-specific chemical shift assignments.

**
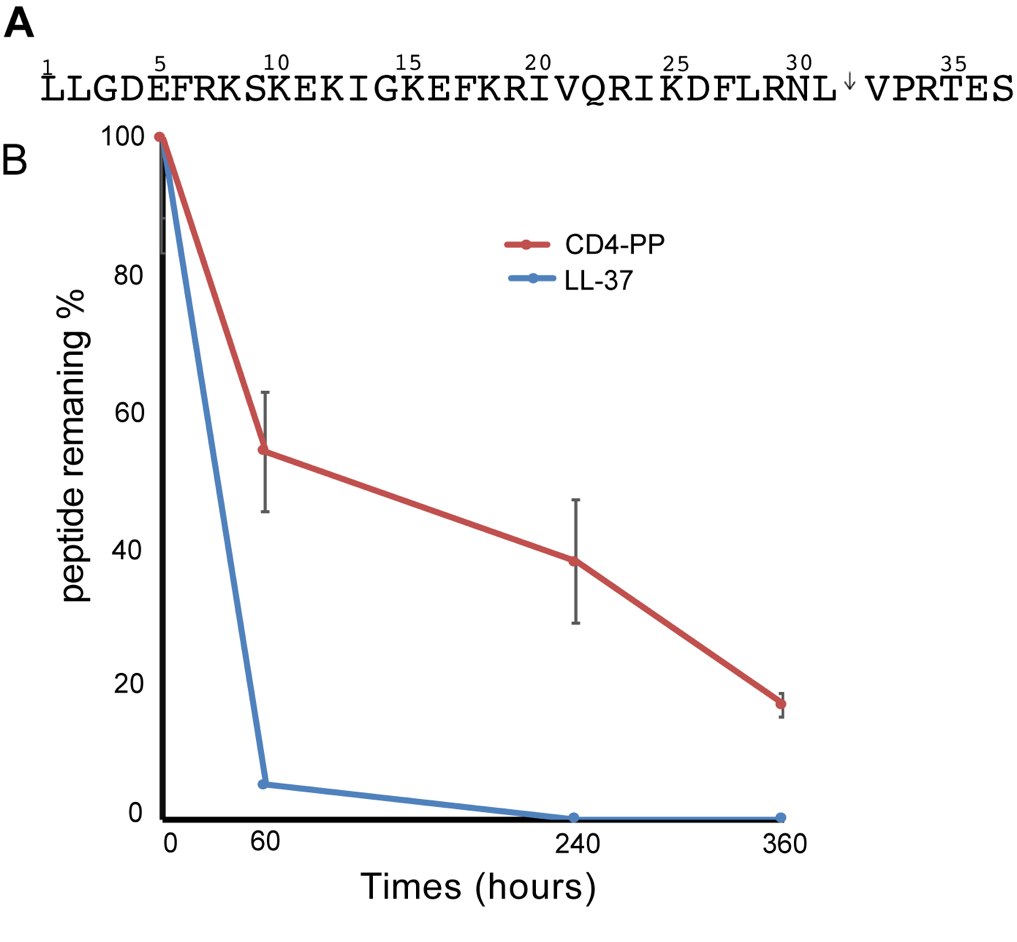

Supplementary Figure S2.  Proteolytic stability of CD4-PP in Aureolysin:** **(a)**Identified cleavage site, dotted arrow between L31 and V32 in LL-37 represent cleavage sites reported in this study  **(b)**Stability of peptides in presence of aureolysin.


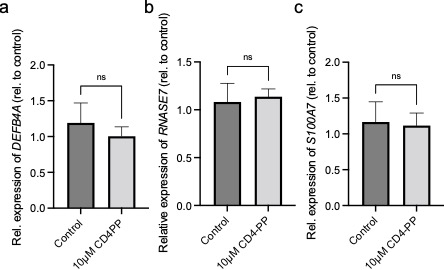


**Supplementary Figure S3. CD4-PP does not induce the expression of other antimicrobial peptides.** Expression of *DEFB4A* (**a**), *RNASE7* (**b**), and *S100A7* (c**)** in uninfected uroepithelial cells, 5637, after stimulation with 10µM of CD4-PP.

**Supplementary Table S1. Sequences, net charge, hydropathicity and molecular weight of peptides**

| **Peptide** | **Sequence** | **Net Charge**  **(at neutral pH)^a^** | **Grand average of hydropathicity** | **Molecular Weight (Expected)^b^** | **Molecular Weight (Observed)^b^** |
| --- | --- | --- | --- | --- | --- |
| CD4-PP | CPGGKRIVKRIKAFLRGPGGKRIVKRIKAFLR | +12 | ­­- 0.309 | 3602.54 | 3603.04 |
| LL-37 | LLGDFFRKSKEKIGKEFKRIVQRIKDFLRNLVPRTES | +6 | - 0.724 | 4493.32 | 4493.98 |

^a^ExPASy ProtParam tool was used to calculate the molecular weight, net charge and hydropathy index (<http://web.expasy.org/protparam/>)

^b^Observed peptides masses presented as (M+1)^+^ have been deconvoluted from (M+2)^2+^, (M+3)^3+^ and (M+4)^4+^ masses.

**Supplementary Table S2. Impact of salts on the minimum inhibitory concentration (MIC) of CD4-PP and L-37.** MICs are in µM.

| Species | Peptides | Tris-buffer  10mM | NaCl  150mM | NH_4_Cl  6 μM | CaCl_2_  2.5 mM | MgCl_2_  1 mM | FeCl_3_  4 μM |
| --- | --- | --- | --- | --- | --- | --- | --- |
| *E. coli* | LL-37 | 0.625 | 5 | 1.25 | >10 | 5 | 2.5 |
|  | CD4-PP | 0.312 | 1,25 | 0.625 | 1,25 | 0.625 | 0.625 |
| *P. aeruginosa* | LL-37 | 1.25 | 5 | 1.25 | >10 | 5 | 2.5 |
|  | CD4-PP | 0.625 | 1.25 | 0.625 | 1.25 | 1.25 | 0.625 |

**Supplementary Table S3. List of genes and their primer sequences.** *ACTB* was used on the housekeeping gene, and the fold change in gene expression was calculated using the 2^-ΔΔCT^ method.

| Gene | Forward Sequence (5’-3’) | Reverse Sequence (5’-3’) |
| --- | --- | --- |
| *ACTB* | AAG AGA GGC ATC CTC ACC CT | TAC ATC GCT GGG GTG TTG |
| *CAMP* | ACC CAG CAG GGC AAA TCT | GAA GGA CGG GCT GGT GAA |
| *CLDN14* | TGT ACC TGG GCT TCA TCT CC | CCT CGC ATT CAC ATT ATT TCC |
| *OCLN* | TTT GTG GGA CAA GGA ACA CA | TCA TTC ACT TTG CCA TTG GAT |
| *CXCL8* | AAG AGA GCT CTG TCT GGA CC | GAT ATT CTC TTG GCC CTT GG |
| *RNASE7* | CAT GGC TGA GTT GCA TCG TTG A | GGA GTC ACA GCA CGA AGA CCA |
| *DEFB4A* | CCC TTT CTG AAT CCG | GAG GGT CTT GTA TCT CCT |
| *S100A7* | CAC CAG ACG TGA TGA CAA | GGC TAT GTC TCC CAG CAA |
